# Data access for the 1,000 Plants (1KP) project

**DOI:** 10.1186/2047-217X-3-17

**Published:** 2014-10-27

**Authors:** Naim Matasci, Ling-Hong Hung, Zhixiang Yan, Eric J Carpenter, Norman J Wickett, Siavash Mirarab, Nam Nguyen, Tandy Warnow, Saravanaraj Ayyampalayam, Michael Barker, J Gordon Burleigh, Matthew A Gitzendanner, Eric Wafula, Joshua P Der, Claude W dePamphilis, Béatrice Roure, Hervé Philippe, Brad R Ruhfel, Nicholas W Miles, Sean W Graham, Sarah Mathews, Barbara Surek, Michael Melkonian, Douglas E Soltis, Pamela S Soltis, Carl Rothfels, Lisa Pokorny, Jonathan A Shaw, Lisa DeGironimo, Dennis W Stevenson, Juan Carlos Villarreal, Tao Chen, Toni M Kutchan, Megan Rolf, Regina S Baucom, Michael K Deyholos, Ram Samudrala, Zhijian Tian, Xiaolei Wu, Xiao Sun, Yong Zhang, Jun Wang, Jim Leebens-Mack, Gane Ka-Shu Wong

**Affiliations:** 1 iPlant Collaborative, Tucson 85721, AZ, USA; 2 Department of Ecology and Evolutionary Biology, University of Arizona, Tucson 85721, AZ, USA; 3 Department of Microbiology, University of Washington, Seattle 98109, WA, USA; 4 BGI-Shenzhen, Bei Shan Industrial Zone, Shenzhen, China; 5 Department of Biological Sciences, University of Alberta, Edmonton T6G 2E9, AB, Canada; 6 Chicago Botanic Garden, Glencoe 60022, IL, USA; 7 Program in Biological Sciences, Northwestern University, Evanston 60208, IL, USA; 8 Department of Computer Science, University of Texas, Austin, TX, 78712, USA; 9 Department of Plant Biology, University of Georgia, Athens, GA, 30602, USA; 10 Department of Biology, University of Florida, Gainesville, FL 32611, USA; 11 Department of Biology, Penn State University, University Park, Pennsylvania, PA, 16801, USA; 12 Département de Biochimie, Centre Robert-Cedergren, Université de Montréal, Succursale Centre-Ville, Montréal, Québec H3C3J7, Canada; 13 CNRS, USR 2936, Station d’ Ecologie Expérimentale du CNRS, Moulis 09200, France; 14 Department of Biological Sciences, Eastern Kentucky University, Richmond, KY, 40475, USA; 15 Florida Museum of Natural History, Gainesville, FL, 32611, USA; 16 Department of Botany, University of British Columbia, Vancouver, BC V6T 1Z4, Canada; 17 Arnold Arboretum of Harvard University, Cambridge, MA, 02138, USA; 18 Botanical Institute, Universität zu Köln, Köln D-50674, Germany; 19 Genetics Institute, University of Florida, Gainesville, FL, 32611, USA; 20 Department of Biology, Duke University, Durham, NC 27708, USA; 21 Department of Zoology, University of British Columbia, Vancouver, BC, V6T 1Z4, Canada; 22 Department of Biodiversity and Conservation, Real Jardín Botánico (RJB-CSIC), 28014 Madrid, Spain; 23 New York Botanical Garden, Bronx, NY, 10458, USA; 24 Systematic Botany and Mycology, University of Munich (LMU), Menzinger Str. 67, 80638 Munich, Germany; 25 Shenzhen Fairy Lake Botanical Garden, The Chinese Academy of Sciences, Shenzhen, Guangdong, 518004, China; 26 Donald Danforth Plant Science Center, St. Louis, MO, 63132, USA; 27 Department of Ecology and Evolutionary Biology, University of Michigan, Ann Arbor, MI, USA; 28 Department of Medicine, University of Alberta, Edmonton, AB, T6G 2E1, Canada

**Keywords:** *Viridiplantae*, Biodiversity, Transcriptomes, Phylogenomics, Interactions, Pathways

## Abstract

The 1,000 plants (1KP) project is an international multi-disciplinary consortium that has generated transcriptome data from over 1,000 plant species, with exemplars for all of the major lineages across the *Viridiplantae* (green plants) clade. Here, we describe how to access the data used in a phylogenomics analysis of the first 85 species, and how to visualize our gene and species trees. Users can develop computational pipelines to analyse these data, in conjunction with data of their own that they can upload. Computationally estimated protein-protein interactions and biochemical pathways can be visualized at another site. Finally, we comment on our future plans and how they fit within this scalable system for the dissemination, visualization, and analysis of large multi-species data sets.

## Introduction

The 1,000 plants (1KP) project is an international multi-disciplinary consortium that has now generated transcriptome data from over 1,000 plant species. One of the goals of our species selection process was to provide exemplars for all of the major lineages across the *Viridiplantae* (green plants), representing approximately one billion years of evolution, including flowering plants, conifers, ferns, mosses and streptophyte green algae. Whereas genomics has long strived for completeness within species (e.g., every gene in the species), we were focused on completeness across an evolutionary clade – obviously not every species, but one representative species for everything at some phylogenetic level (e.g., one species per family, and perhaps more than one species when the family is especially large). Because many of our species had never been subjected to large-scale sequencing, 2 gigabases (Gb) of data per sample was sufficient to increase the number of plant genes by approximately 100-fold in comparison to the totality of the public databases.

The 1KP project began as a public-private partnership, with 75% of the funding provided by the Government of Alberta and 25% by Musea Ventures. Significant in-kind contributions were provided by BGI-Shenzhen in the form of reduced sequencing costs and by the NSF-funded iPlant collaborative [1] in the form of computational informatics support. Many plant scientists from around the world were involved in the collection of live tissue samples and in the extraction of RNA. Additional computing resources were provided by Compute Canada and by the China National GeneBank. Despite the constraints of this funding model, we released our data (on a collaborative basis) to scientists who approached us with goals that did not compete with ours. For the general community, access was provided through a BLAST portal [2].

We believed that there would be intrinsic value in data of this nature that is beyond our imagination. But for the initial publication, we agreed on two objectives. Firstly, by adopting a phylogenomics approach we hoped to resolve many of the lingering uncertainties in species relationships, especially in the early lineages of streptophyte green algae and land plants, where previous analyses were based on comparatively sparse taxonomic densities. And secondly, despite the limitations of these data, we hoped to identify some of the gene changes associated with the major innovations in *Viridiplantae* evolution, such as multicellularity, transitions from marine to freshwater or terrestrial environments, maternal retention of zygotes and embryos, complex life history involving haploid and diploid phases, vascular systems, seeds and flowers.

Our RNA extraction protocols [3] and our RNA-Seq transcriptome assembly algorithms [4] have already been published. Here, we are publishing the second of two linked papers. The first is a review of the state-of-knowledge for *Viridiplantae* species relationships and our initial foray into the phylogenomics on a subset of 1KP [5]. The other is a description of the websites that we created in order to provide access to the data (from raw reads to computed results), visualize the results, and perform custom analyses in conjunction with external data that the users can upload. An initial gene annotation is also provided, which focuses on the functional relationships between proteins and their associated metabolites.

## Review

### Access to raw and processed data

Our initial phylogenomics effort used sequences from multiple sources. They include transcriptomes from 1KP representing 85 species, transcriptomes from other sources representing 7 species, and genomes representing an additional 11 species. A summary of these data sources is given in Table 1. We submitted all of the unassembled reads from the 1KP transcriptomes to the Short Reads Archive (SRA) under project accession PRJEB4921 “1000 Plant (1KP) Transcriptome: The Pilot Study.” Note that, with the exception of *Eschscholzia californica*, we sequenced only one sample per species.

**Table 1:** Data sources for phylogenomics analyses

**Species**	**Type**	**Accession**	**iPlant ID**
*Arabidopsis thaliana*	genome	n/a	n/a
*Brachypodium distachyon*	genome	n/a	n/a
*Carica papaya*	genome	n/a	n/a
*Medicago truncatula*	genome	n/a	n/a
*Oryza sativa*	genome	n/a	n/a
*Physcomitrella patens*	genome	n/a	n/a
*Populus trichocarpa*	genome	n/a	n/a
*Selaginella moellendorffii*	genome	n/a	n/a
*Sorghum bicolor*	genome	n/a	n/a
*Vitis vinifera*	genome	n/a	n/a
*Zea mays*	genome	n/a	n/a
*Aquilegia formosa*	meta-assembly	PlantGDB	AQUI
*Cycas rumphii*	meta-assembly	SRX022306, SRX022215	CYCA
*Liriodendron tulipifera*	meta-assembly	PRJNA46857	LIRI
*Persea americana*	meta-assembly	PRJNA46857	PERS
*Pinus taeda*	meta-assembly	PRJNA79733	PINU
*Pteridium aquilinum*	meta-assembly	PRJNA48473	PTER
*Zamia vazquezii*	meta-assembly	PRJNA46857	ZAMI
*Acorus americanus*	OneKP meta-assembly	ERR364395, PRJNA46857	ACOR
*Amborella trichopoda*	OneKP meta-assembly	ERR364329, PRJNA46857	AMBO
*Catharanthus roseus*	OneKP meta-assembly	ERR364390, PRJNA79951, PRJNA236160	CATH
*Eschscholzia californica*	OneKP meta-assembly	ERR364338, ERR364335, ERR364336, ERR364337, ERR364334, SRX002988, SRX002987, PlantGDB	ESCH
*Ginkgo biloba*	OneKP meta-assembly	ERR364401, PlantGDB	GINK
*Nuphar advena*	OneKP meta-assembly	ERR364330, PRJNA46857	NUPH
*Ophioglossum petiolatum*	OneKP meta-assembly	ERR364410, SRX666586	OPHI
*Saruma henryi*	OneKP meta-assembly	ERR364383, PRJNA46857	SARU
*Welwitschia mirabilis*	OneKP meta-assembly	ERR364404, PRJNA46857	WELW
*Allamanda cathartica*	OneKP	ERR364389	MGVU
*Angiopteris evecta*	OneKP	ERR364409	NHCM
*Anomodon attenuatus*	OneKP	ERR364349	QMWB
*Bazzania trilobata*	OneKP	ERR364415	WZYK
*Boehmeria nivea*	OneKP	ERR364387	ACFP
*Bryum argenteum*	OneKP	ERR364348	JMXW
*Cedrus libani*	OneKP	ERR364342	GGEA
*Ceratodon purpureus*	OneKP	ERR364350	FFPD
*Chaetosphaeridium globosum*	OneKP	ERR364369	DRGY
*Chara vulgaris*	OneKP	ERR364366	CHAR
*Chlorokybus atmophyticus*	OneKP	ERR364371	AZZW
*Colchicum autumnale*	OneKP	ERR364397	NHIX
*Coleochaete irregularis*	OneKP	ERR364367	QPDY
*Coleochaete scutata*	OneKP	ERR364368	VQBJ
*Cosmarium ochthodes*	OneKP	ERR364376	STKJ
*Cunninghamia lanceolata*	OneKP	ERR364340	OUOI
*Cyathea (Alsophila) spinulosa*	OneKP	ERR364412	GANB
*Cycas micholitzii*	OneKP	ERR364405	XZUY
*Cylindrocystis brebissonii*	OneKP	ERR364378	YOXI
*Cylindrocystis cushleckae*	OneKP	ERR364373	JOJQ
*Dendrolycopodium obscurum*	OneKP	ERR364346	XNXF
*Dioscorea villosa*	OneKP	ERR364396	OCWZ
*Diospyros malabarica*	OneKP	ERR364339	KVFU
*Entransia fimbriata*	OneKP	ERR364372	BFIK
*Ephedra sinica*	OneKP	ERR364402	VDAO
*Equisetum diffusum*	OneKP	ERR364408	CAPN
*Gnetum montanum*	OneKP	ERR364403	GTHK
*Hedwigia ciliata*	OneKP	ERR364352	YWNF
*Hibiscus cannabinus*	OneKP	ERR364388	OLXF
*Houttuynia cordata*	OneKP	ERR364332	CSSK
*Huperzia squarrosa*	OneKP	ERR364407	GAON
*Inula helenium*	OneKP	ERR364393	AFQQ
*Ipomoea purpurea*	OneKP	ERR364392	VXKB
*Juniperus scopulorum*	OneKP	ERR364341	XMGP
*Kadsura heteroclita*	OneKP	ERR364331	NWMY
*Klebsormidium subtile*	OneKP	ERR364370	FQLP
*Kochia scoparia*	OneKP	ERR364385	WGET
*Larrea tridentata*	OneKP	ERR364386	UDUT
*Leucodon brachypus*	OneKP	ERR364353	ZACW
*Marchantia emarginata*	OneKP	ERR364417	TFYI
*Marchantia polymorpha*	OneKP	ERR364416	JPYU
*Mesostigma viride*	OneKP	ERR364365	KYIO
*Mesotaenium endlicherianum*	OneKP	ERR364377	WDCW
*Metzgeria crassipilis*	OneKP	ERR364359	NRWZ
*Monomastix opisthostigma*	OneKP	ERR364362	BTFM
*Mougeotia* sp.	OneKP	ERR364374	ZRMT
*Nephroselmis pyriformis*	OneKP	ERR364363	ISIM
*Netrium digitus*	OneKP	ERR364379	FFGR
*Nothoceros aenigmaticus*	OneKP	ERR364356	DXOU
*Nothoceros vincentianus*	OneKP	ERR364357	TCBC
*Penium margaritaceum*	OneKP	ERR364382	AEKF
*Podophyllum peltatum*	OneKP	ERR364384	WFBF
*Polytrichum commune*	OneKP	ERR364413	SZYG
*Prumnopitys andina*	OneKP	ERR364343	EGLZ
*Pseudolycopodiella caroliniana*	OneKP	ERR364345	UPMJ
*Psilotum nudum*	OneKP	ERR364411	QVMR
*Pyramimonas parkeae*	OneKP	ERR364361	TNAW
*Rhynchostegium serrulatum*	OneKP	ERR364355	JADL
*Ricciocarpos natans*	OneKP	ERR364358	WJLO
*Rosmarinus officinalis*	OneKP	ERR364391	FDMM
*Rosulabryum* cf. *capillare*	OneKP	ERR364351	XWHK
*Roya obtusa*	OneKP	ERR364380	XRTZ
*Sabal bermudana*	OneKP	ERR364400	HWUP
*Sarcandra glabra*	OneKP	ERR364333	OSHQ
*Sciadopitys verticillata*	OneKP	ERR364344	YFZK
*Selaginella stauntoniana*	OneKP	ERR364347	ZZOL
*Smilax bona-nox*	OneKP	ERR364398	MWYQ
*Sphaerocarpos texanus*	OneKP	ERR364360	HERT
*Sphagnum lescurii*	OneKP	ERR364414	GOWD
*Spirogyra* sp.	OneKP	ERR364375	HAOX
*Spirotaenia minuta*	OneKP	ERR364381	NNHQ
*Tanacetum parthenium*	OneKP	ERR364394	DUQG
*Taxus baccata*	OneKP	ERR364406	WWSS
*Thuidium delicatulum*	OneKP	ERR364354	EEMJ
*Uronema* sp.	OneKP	ERR364364	ISGT
*Yucca filamentosa*	OneKP	ERR364399	ICNN

Meta-assembly refers to a transcriptome assembled from more than one sequenced sample. Some of these were a combination of 1KP and other data; some were entirely non-1KP. Accession numbers (SRA or otherwise) are given for all of the transcriptomes that we used.

To make it easier for others to reproduce our phylogenomics analyses, we are releasing our intermediate computations, not just the final results. Everything is hosted at the iPlant Data Store, a high performance, large capacity, distributed storage system. The contents include transcriptome assemblies, putative coding sequences, orthogroups (i.e*.*, from the 11 reference genomes), as well as gene and species trees with related sequence alignments. There are quite a lot of files and their total sizes are not negligible; so before users begin to download these files, we suggest that they consult Table 2 for a description of what to expect.

**Table 2:** Number and size of data files on websites

**File count**	**Median size (Mb)**	**Average size (Mb)**	**Largest size (Mb)**	**Total size (Mb)**	**Similar directories**	**iPlant directory name**
68,253	0.0	0.3	481.1	23,116.6		onekp_pilot
48,053	0.0	0.3	481.1	14,956.7		onekp_pilot/orthogroups
19,220	0.1	0.7	243.8	13,276.5		onekp_pilot/orthogroups/alignments
9,610	0.1	0.3	79.8	3,289.6		onekp_pilot/orthogroups/alignments/FAA
9,610	0.2	1.0	243.8	9,986.9		onekp_pilot/orthogroups/alignments/FNA
28,833	0.0	0.1	481.1	1,680.2		onekp_pilot/orthogroups/gene_trees
9,611	0.0	0.1	481.1	583.3		onekp_pilot/orthogroups/gene_trees/FAA
9,610	0.0	0.0	0.5	102.2		onekp_pilot/orthogroups/gene_trees/FAA/trees
19,222	0.0	0.1	458.0	1,096.8		onekp_pilot/orthogroups/gene_trees/FNA
9,611	0.0	0.1	458.0	556.6		onekp_pilot/orthogroups/gene_trees/FNA/12_codon
9,610	0.0	0.0	0.5	98.5		onekp_pilot/orthogroups/gene_trees/FNA/12_codon/trees
9,611	0.0	0.1	439.1	540.3		onekp_pilot/orthogroups/gene_trees/FNA/all_codon
9,610	0.0	0.0	0.5	101.2		onekp_pilot/orthogroups/gene_trees/FNA/all_codon/dna_tree
19,919	0.0	0.2	175.2	3,468.8		onekp_pilot/phylogenetic_analysis
2,556	0.1	0.1	1.0	292.7		onekp_pilot/phylogenetic_analysis/alignments
852	0.0	0.0	0.3	41.8		onekp_pilot/phylogenetic_analysis/alignments/FAA
852	0.1	0.1	1.0	125.5		onekp_pilot/phylogenetic_analysis/alignments/FNA
852	0.1	0.1	0.9	125.4		onekp_pilot/phylogenetic_analysis/alignments/FNA2AA
17,197	0.0	0.1	0.4	1,827.3		onekp_pilot/phylogenetic_analysis/gene_trees
1,704	0.0	0.1	0.4	238.3		onekp_pilot/phylogenetic_analysis/gene_trees/FAA
2	0.3	0.1	0.4	0.3	852	onekp_pilot/phylogenetic_analysis/gene_trees/FAA/raxmlboot.####
1,704	0.0	0.1	0.4	238.3		onekp_pilot/phylogenetic_analysis/gene_trees/FNA
2	0.3	0.1	0.4	0.3	852	onekp_pilot/phylogenetic_analysis/gene_trees/FNA/raxmlboot.####
3,408	0.0	0.1	0.4	476.7		onekp_pilot/phylogenetic_analysis/gene_trees/FNA2AA
2	0.3	0.1	0.4	0.3	852	onekp_pilot/phylogenetic_analysis/gene_trees/FNA2AA/raxmlboot.####
2	0.3	0.1	0.4	0.3	852	onekp_pilot/phylogenetic_analysis/gene_trees/FNA2AA/raxmlboot.####.c1c2
10,381	0.0	0.1	0.4	874.0		onekp_pilot/phylogenetic_analysis/gene_trees/filtered
2,548	0.0	0.1	0.4	169.3		onekp_pilot/phylogenetic_analysis/gene_trees/filtered/FAA
1	0.0	0.0	0.0	0.0	852	onekp_pilot/phylogenetic_analysis/gene_trees/filtered/FAA/raxmlboot.####.f25
1	0.2	0.1	0.4	0.2	852	onekp_pilot/phylogenetic_analysis/gene_trees/filtered/FAA/raxmlboot.####.filterlen33
852	0.0	0.0	0.0	3.8		onekp_pilot/phylogenetic_analysis/gene_trees/filtered/FNA
1	0.0	0.0	0.0	0.0	852	onekp_pilot/phylogenetic_analysis/gene_trees/filtered/FNA/raxmlboot.####.f25
6,980	0.0	0.1	0.4	700.9		onekp_pilot/phylogenetic_analysis/gene_trees/filtered/FNA2AA
2	0.3	0.1	0.4	0.3	852	onekp_pilot/phylogenetic_analysis/gene_trees/filtered/FNA2AA/raxmlboot.####.GAMMA.2
2	0.3	0.1	0.4	0.3	852	onekp_pilot/phylogenetic_analysis/gene_trees/filtered/FNA2AA/raxmlboot.####.c1c2.GAMMA.2
1	0.0	0.0	0.0	0.0	852	onekp_pilot/phylogenetic_analysis/gene_trees/filtered/FNA2AA/raxmlboot.####.c1c2.f25
1	0.0	0.0	0.0	0.0	852	onekp_pilot/phylogenetic_analysis/gene_trees/filtered/FNA2AA/raxmlboot.####.f25
2	0.2	0.1	0.4	0.2	844	onekp_pilot/phylogenetic_analysis/gene_trees/filtered/FNA2AA/raxmlboot.####.filterlen33
1	0.3	0.3	0.4	0.3	180	onekp_pilot/phylogenetic_analysis/gene_trees/filtered/FNA2AA/raxmlboot.####.filtered25.GAMMA.2
166	0.0	8.1	175.2	1,348.8		onekp_pilot/phylogenetic_analysis/species_level
50	15.0	27.0	175.2	1,348.1		onekp_pilot/phylogenetic_analysis/species_level/alignments
15	14.7	14.3	58.3	214.2		onekp_pilot/phylogenetic_analysis/species_level/alignments/FAA
35	29.4	32.4	175.2	1,133.9		onekp_pilot/phylogenetic_analysis/species_level/alignments/FNA
116	0.0	0.0	0.0	0.6		onekp_pilot/phylogenetic_analysis/species_level/trees
276	10.0	17.0	157.4	4,691.1		onekp_pilot/taxa
3	9.7	17.0	157.4	51.0	92	onekp_pilot/taxa/####-############
1	30.8	17.0	157.4	36.0	92	onekp_pilot/taxa/####-############/assemblies
2	9.7	7.5	45.2	15.0	92	onekp_pilot/taxa/####-############/translations
5	0.0	0.0	0.0	0.1		onekp_pilot/tools
**File count**	**Median size (Mb)**	**Average size (Mb)**	**Largest size (Mb)**	**Total size (Mb)**	**Similar directories**	**Contents at SRA (PRJEB4921)**
178	1,915.0	2,045.5	3,371.0	364,100.0		total of all short reads -- uncompressed, but downloads are compressed to a quarter of these sizes
2	1,915.0	2,045.5	3,371.0	4,091.0	89	expecting per sample -- uncompressed, but downloads are compressed to a quarter of these sizes

In some instances, users will find many directories with similar names, as indicated in this table by hash (#) marks. The total number of directories is given in the preceding column.

At the simplest level, anonymous downloads are permitted from a designated area of the iPlant Data Store [6]. However, much greater functionality is available through the iPlant resources that we describe in the following sections.

### Visualization and custom analyses

To take full advantage of the iPlant computational infrastructure, it is necessary to first register at [7]. Accounts are free, and in addition to 1KP data, users will find high performance computing and cloud-based services. Multiple access modalities are supported: anonymous and secure web interfaces, desktop clients and high-speed command lines. However, we feel that for most users the best option is the iPlant discovery environment (DE), a web-based interface that provides users with high-performance computing resources and data storage. Most contemporary web browsers are supported, including Safari v. 6.1, Firefox v. 24, and Chrome v. 34. The caveat is that some of these functionalities (see below) require Java 1.6.

To guide users through its resources, iPlant is constantly producing new tutorials and teaching materials, including live and recorded webinars. The full catalog can be found at [8]. Here, we describe the new resources specifically created for 1KP.

#### Discovery environment (DE)

**Figure 1:**
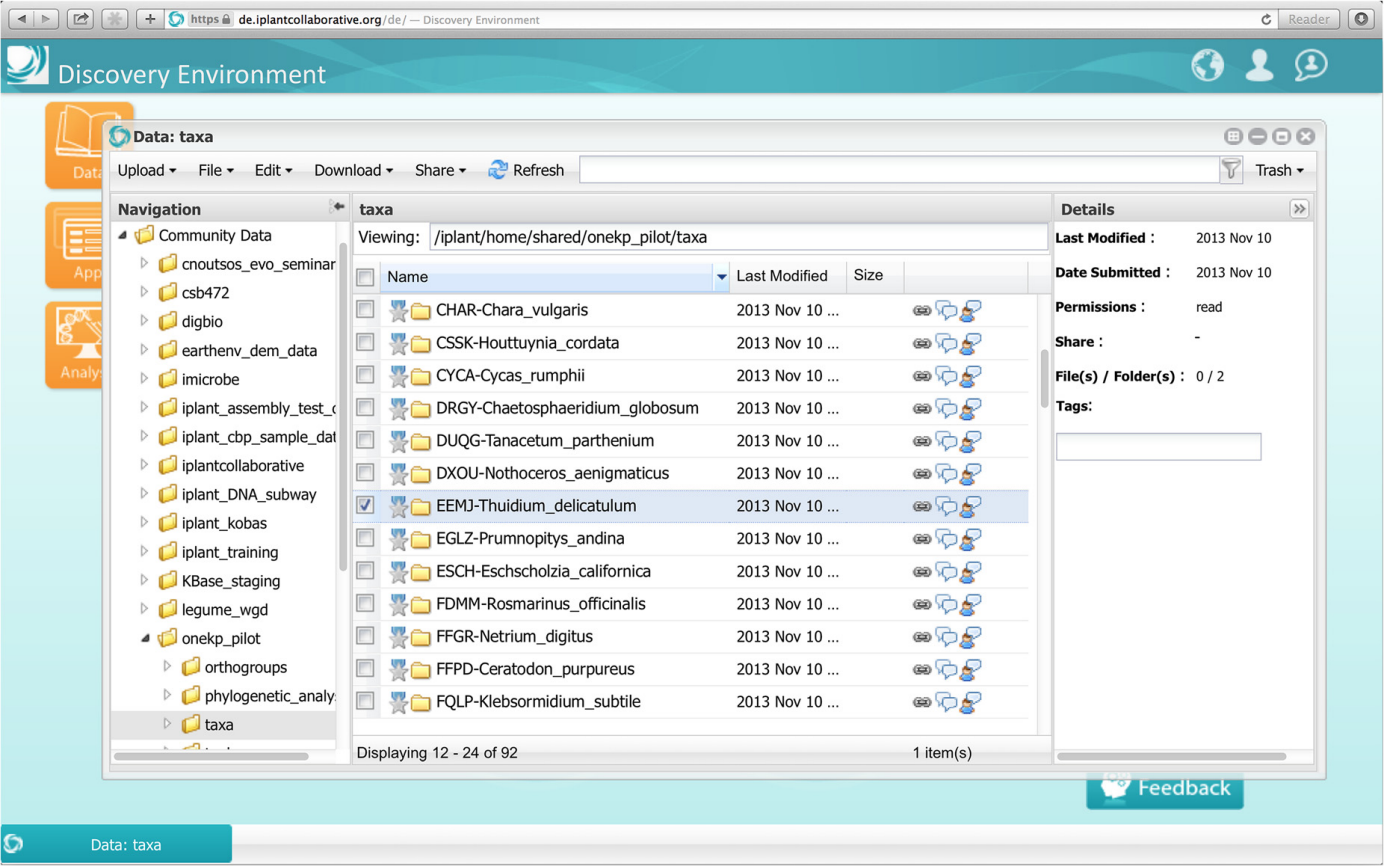
iPlant DE data window.

For access to the 1KP files, users should visit [9] and search for a folder called *Community Data/onekp_pilot* Figure 1.

**Figure 2:**
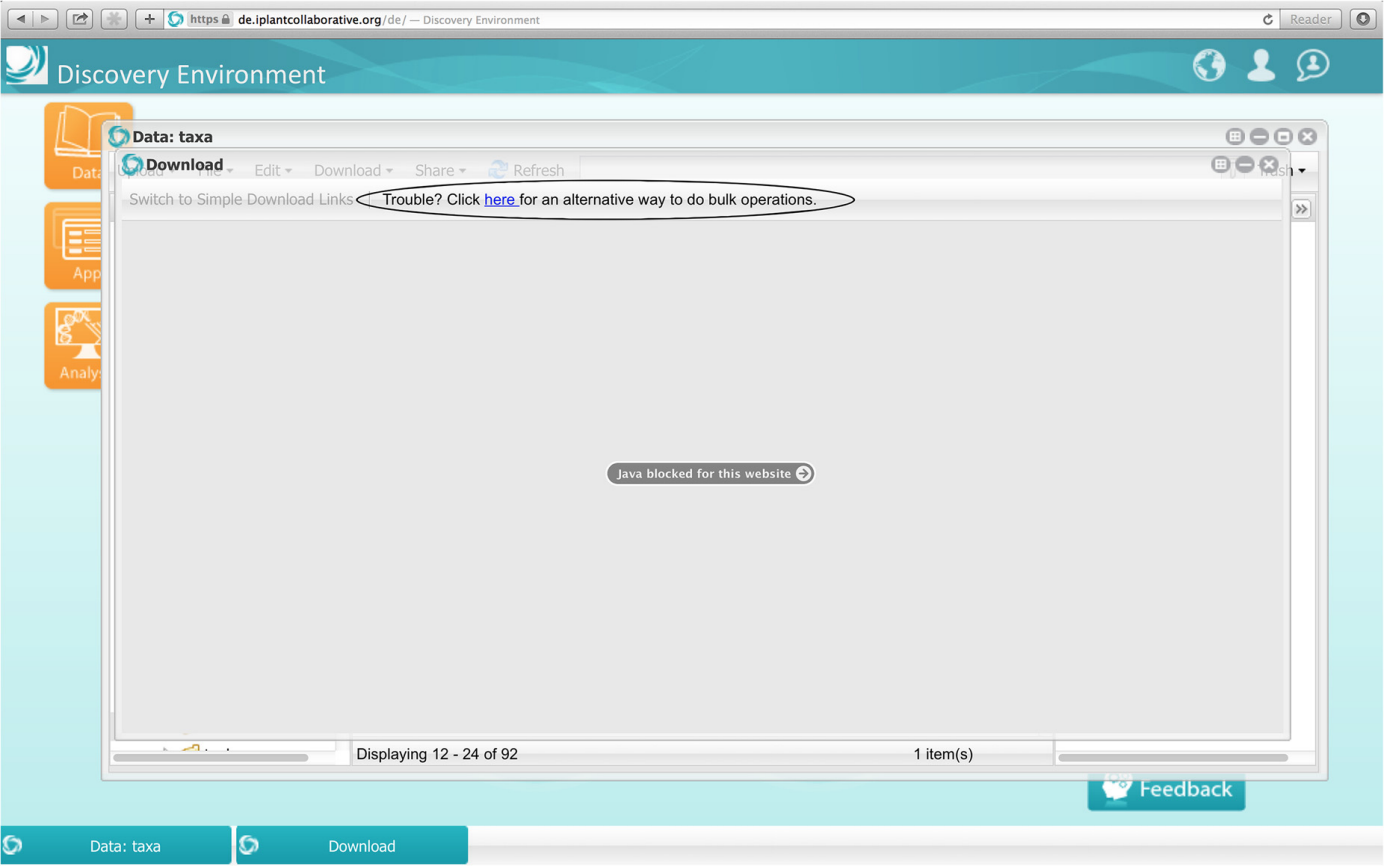
**Bulk download window if Java is disabled.** Click on the circled link to access the instructions to install and configure an iDrop desktop.

From the data window it is possible to download individual files or perform bulk downloads of multiple files and directories through a Java plugin. Note that for security reasons, some operating systems will not allow users to run Java applets. In this instance, a window will pop up to tell the user that there is a problem, and the user should follow the instructions that are given to configure an iDrop desktop [10] Figure 2.

**Figure 3:**
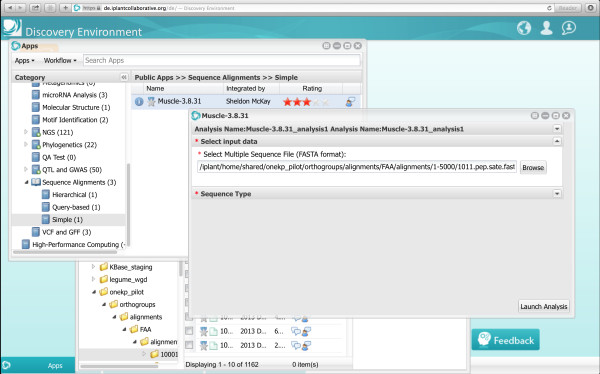
**Realigning a group of sequences using *Muscle.***

It is possible to perform analyses directly in the DE using any of the 1KP files as input; for example, users can re-compute the sequence alignments and gene trees using different algorithms and parameters [11] Figure 3. More generally, users can select from a variety of applications in the Apps catalogue, which is constantly growing, and includes many popular bioinformatics tools for large-scale phylogenetics, genome-wide associations and next generation sequence analyses.

**Figure 4:**
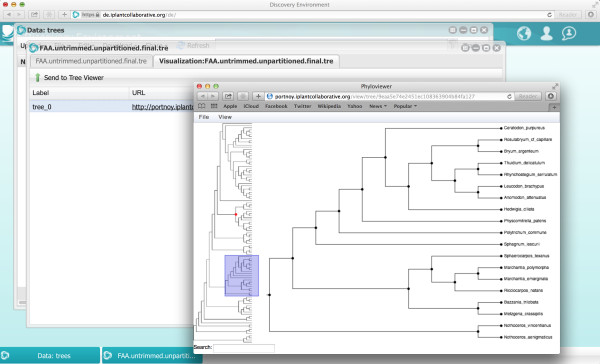
***Phylozoom *** **display of 1KP species phylogeny.**

Species and gene trees can be explored with the iPlant tree viewer, *Phylozoom*, a newly developed web-based phylogenetic tree viewer that supports trees with hundreds of thousand leaves and allows for semantic zooming Figure 4. To access the tree viewer, users need only click on a tree file. This will open a preview window with two tabs: one for the tree’s newick string (a format for graph-theoretical trees as defined at [12]) and another for the web link that opens a window to the tree display. Notice that pop-ups must be enabled on the user’s browser.

To zoom in and expand the collapsed clades, click on the node of interest. To zoom out, click and drag the tree figure to the left. To zoom out completely, click the space bar. The web address is a unique identifier that can be shared with others to let them to visualize the tree.

For more advanced users wanting to perform more complicated procedures, iPlant capabilities are available from a command line. It is based on the integrated rule-oriented data system (iRODS) [13]. All the user has to do is install a command line utility, *icommands*, which mimics UNIX and enables high-speed parallel data transfers. Instructions are available at [14].

#### Interactions and pathways

**Figure 5:**
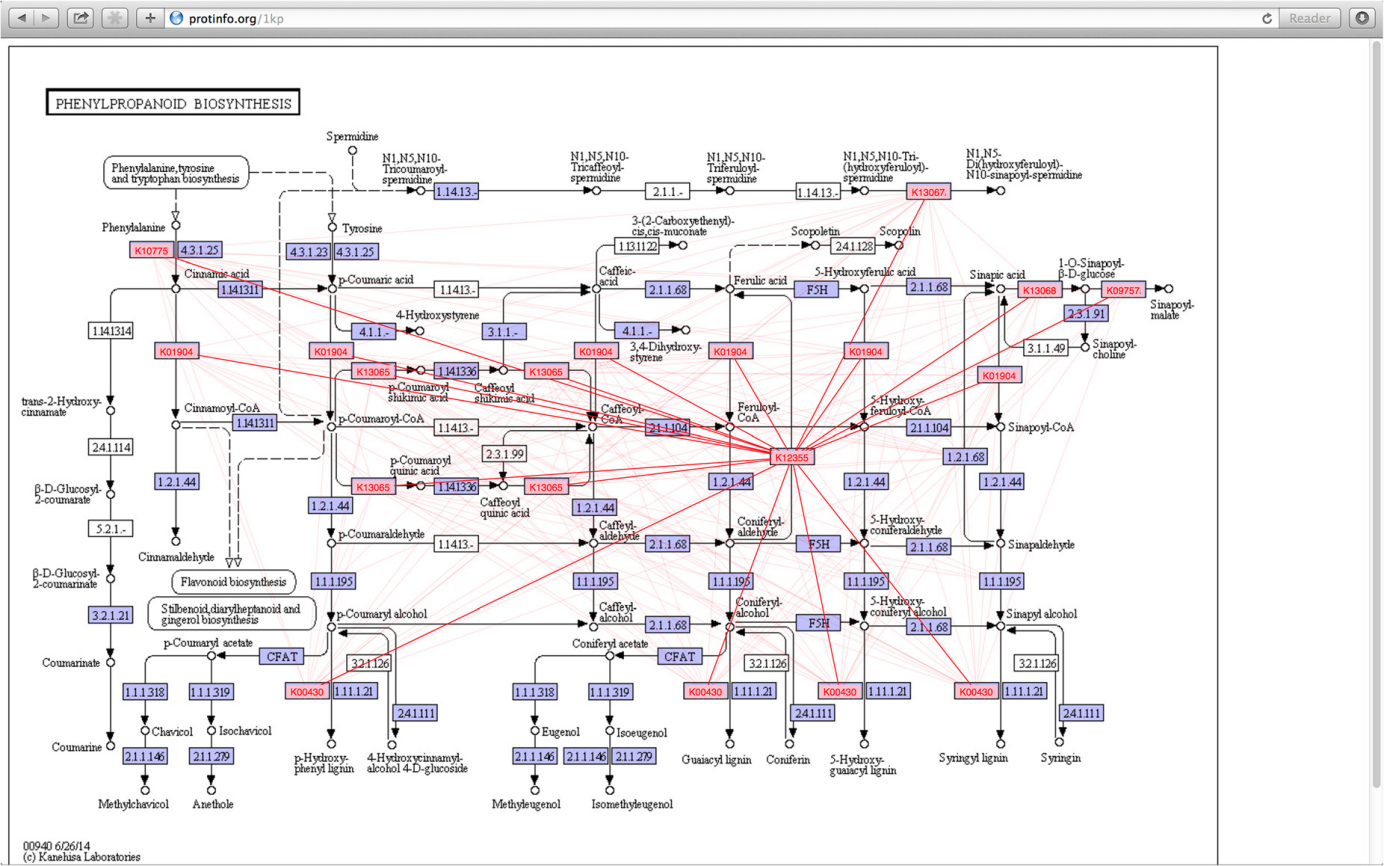
**Phenylpropanoid synthesis pathway for *Colchicum autumnale.*** Labelled rectangles are proteins. Small circles are metabolites. Black lines show the KEGG pathway. Red lines show the BioGRID interactions emanating from protein (K12355), which was interactively selected. A right-click on the protein will display the inferred function and a link to the sequence(s).

In addition to the tree-based species and gene relationships at the iPlant site, functional relationships between proteins and their associated metabolites are available from the Computational Biology Group at the University of Washington, developers of CANDO [15]. Sequence similarity-based methods are used to map 1KP proteins to curated repositories of protein-protein interactions (i.e., BioGRID [16]) and biochemical pathways (i.e., Kyoto Encylopedia of Genes and Genomes [KEGG] [17]). The user can select any metabolic pathway defined by KEGG and, within this context, see all the 1KP proteins from their chosen species, with functional annotations inferred from KEGG. This website is at [18] Figure 5.

Note that, over the course of this project, there have been many improvements in the transcriptome assemblies. The phylogenomics work (now being published) was done with the SOAPdenovo algorithm. A second assembly was subsequently done with the newer SOAPdenovo-trans algorithm, which we incorporated into the newer interactions and pathways work. However, both sets of assemblies are available through the iPlant data store.

## Conclusions

The rest of the 1KP data will be released, on much the same platform, along with our analyses of all one thousand species. Our scientific objectives are given at [19]. We have always been open about our intentions, because we wanted to avoid conflict among the scientists who were already working with 1KP and offer early pre-publication access to other non-competing scientists. As soon as we see a draft of a paper, we track its progress through the review process at [20]. Some of these papers have already been published, and more than a few required years of follow-up experiments, resulting for example in fundamental discoveries for molecular evolution [21] and (surprisingly) new tools for mammalian neurosciences [22].

Many of these studies were not anticipated when 1KP was conceived. We only knew that, just as there was value in sequencing every gene in a genome, despite not knowing *a priori* what many of the genes might do, there would be value in sequencing across an ancient and ecologically dominant clade, even when many of the species have no obvious economic or scientific value that would justify a genome sequencing effort. Transcriptomes were a less expensive way to explore plant diversity, and demonstrate value beyond the obvious species.

## Abbreviations

1KP: 1,000 Plants project; DE: Discovery Environment; KEEG: Kyoto Encyclopedia of Genes and Genomes; NSF: National Science Foundation; SRA: Short Reads Archive.

## Competing interests

The authors declare that they have no competing interests.

## Authors’ contributions

CWD, BRR, NWM, SWG, S Ma, BS, MM, DES, PSS, CR, LP, JAS, LD, DWS, JCV, TC, TMK, MR, RSB, MKD, and JLM collected the plant samples. NM, NJW, S Mi, NN, TW, SA, MB, JGB, MAG, EW, JPD, CWD, BR, HP, BRR, and JLM performed the phylogenomic analyses. NM, LHH, ZY, and EJC setup and maintained web-resources used to communicate data. LHH and RS performed the protein and KEGG pathway analyses. EJC, ZT, XW, XS, YZ, JW, and GKW generated the sequence data. GKW and JLM designed and oversaw the research. All authors read and approved the final manuscript.
